# Supplementary material for: Assembly assay identifies a critical region of human fibrillin-1 required for 10–12 nm diameter microfibril biogenesis
Source: PLoS One. 2021 Mar 18;16(3):e0248532. doi: 10.1371/journal.pone.0248532 (PMC7971562; doi:10.1371/journal.pone.0248532)
Supplement: S1 Raw images — (PDF) [file pone.0248532.s003.pdf]

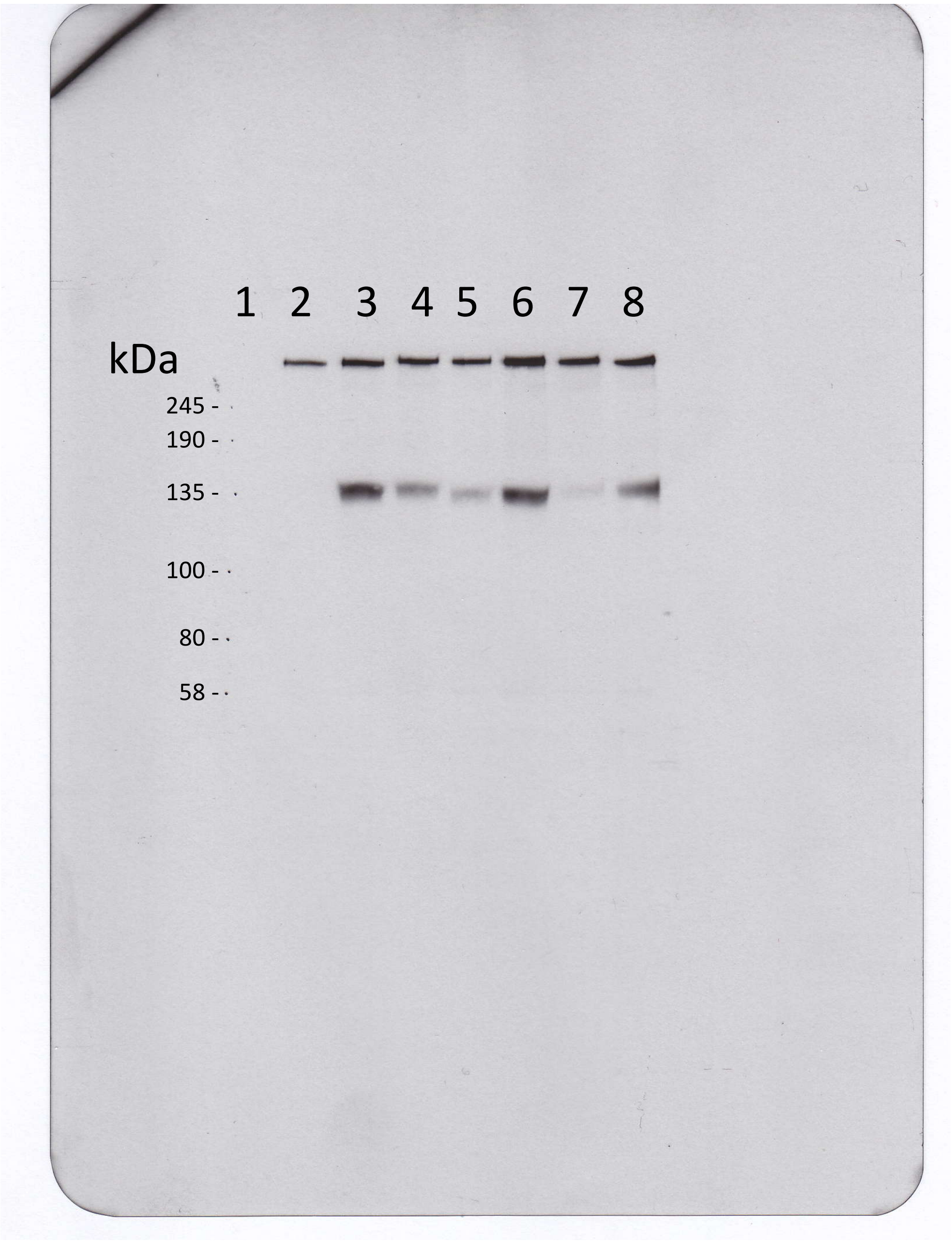

Fig. 2, panel C, cMFS blot

Lanes:

1. Size markers (NEB P7712)
2. untransfected MSU-1.1 control
3. WT
4. Y1101C
5. D1113G
6. D1115G
7. G1127S
8. N1382S

Image captured with a Canon desktop scanner

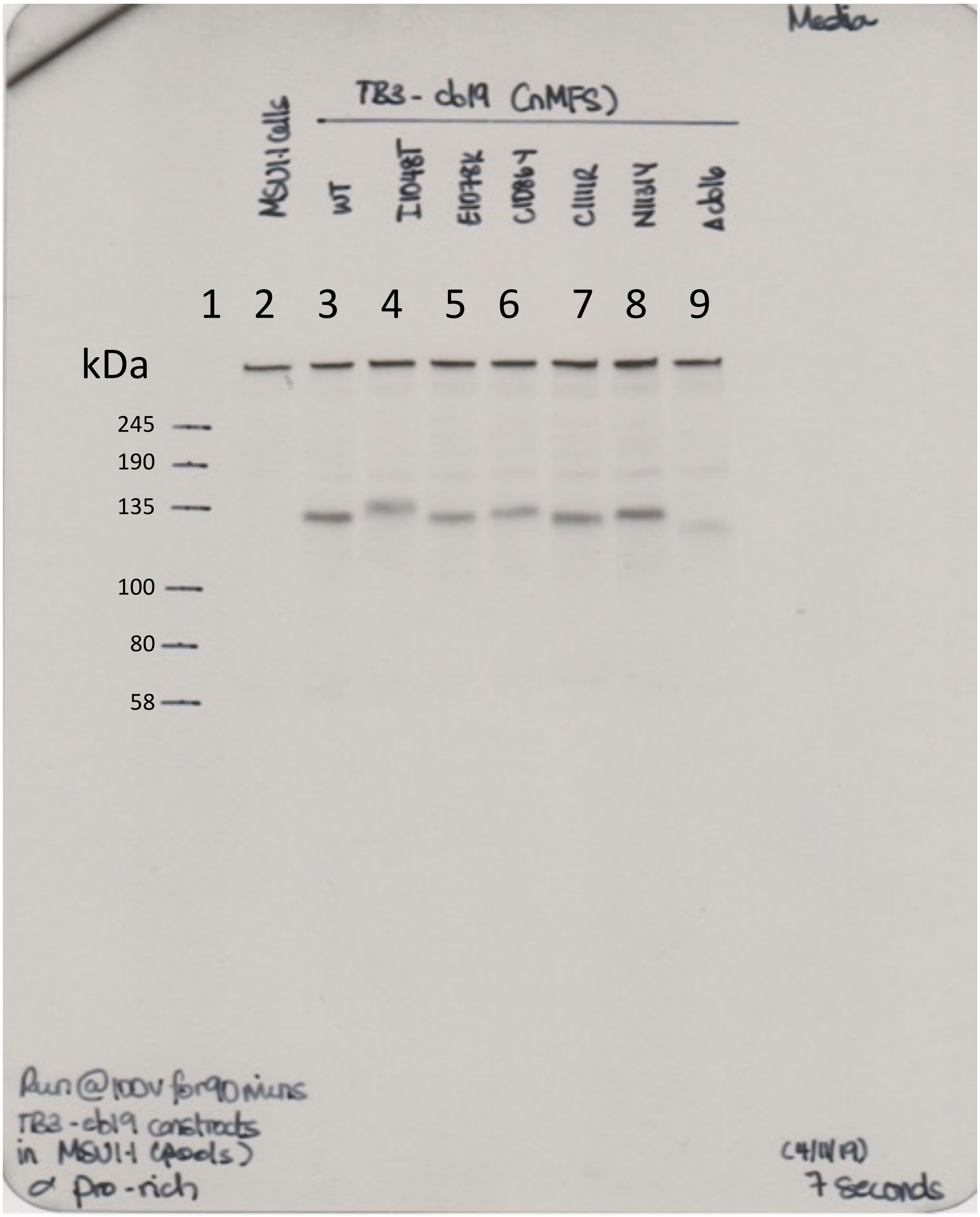

Fig. 2, panel C, nMFS blot

Lanes:

1. Size markers (NEB P7712)
2. untransfected MSU-1.1 control
3. WT
4. I1048T
5. E1073K (*mislabeled as E1078K)
6. C1086Y
7. C1111R
8. N1131Y
9. ∆cbEGF16

Image captured with a Canon desktop scanner

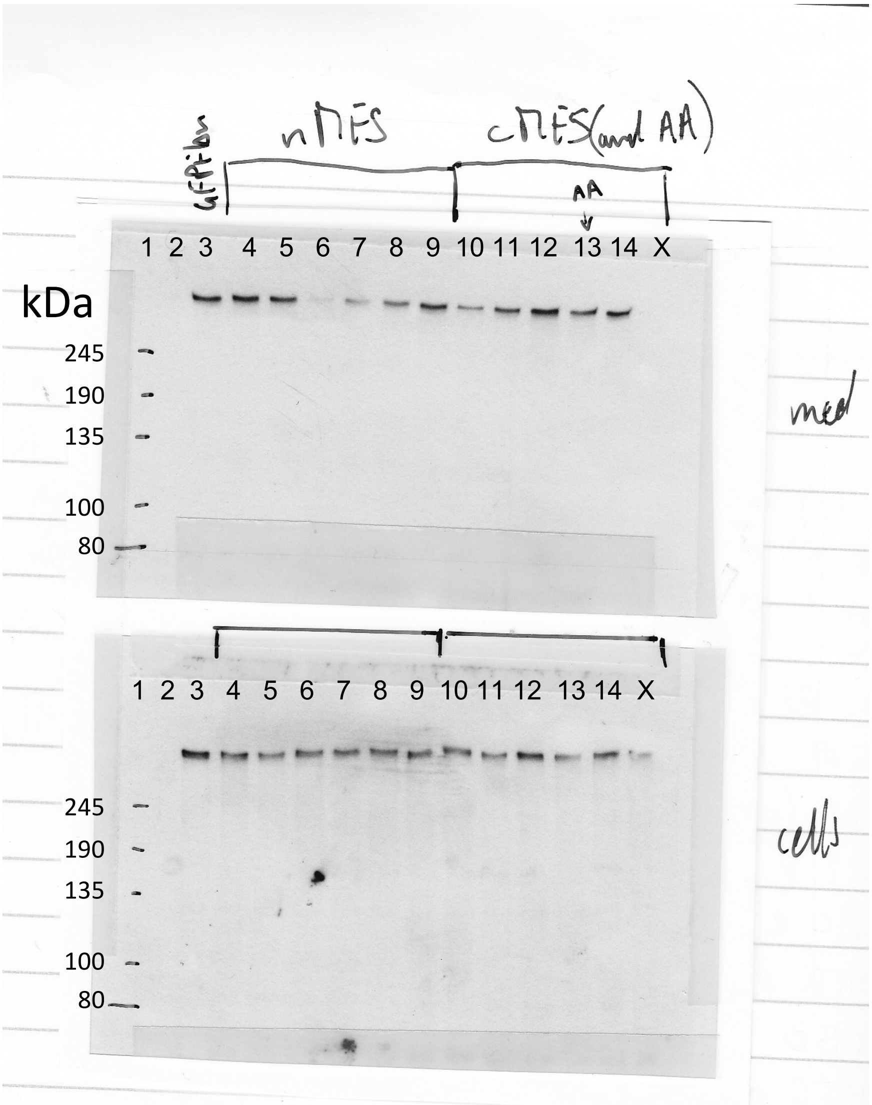

Fig. 3 blots

Lanes:

1. Size markers (NEB P7712)
2. pcDNA
3. GFPFbn WT
4. I1048T
5. E1073K
6. C1086y
7. C1111R
8. N1131Y
9. ∆cbEGF16
10. Y1101C
11. D1113G
12. D1115G
13. G1127S
14. N1382S

X. C1720Y (domain TB5 MFS mutant, not used in paper)

Image scanned from lab notebook with a Canon desktop scanner
